# How “Berry Phase” Analysis of Non-Adiabatic Non-Hermitian Systems Reflects Their Geometry

**DOI:** 10.3390/e25020390

**Published:** 2023-02-20

**Authors:** Chris Jeynes

**Affiliations:** Ion Beam Centre, University of Surrey, Guildford GU2 7XH, UK; c.jeynes@surrey.ac.uk

**Keywords:** open systems, irreversibility, second law of thermodynamics

## Abstract

There is currently great interest in systems represented by non-Hermitian Hamiltonians, including a wide variety of real systems that may be dissipative and whose behaviour can be represented by a “phase” parameter that characterises the way “exceptional points” (singularities of various sorts) determine the system. These systems are briefly reviewed here with an emphasis on their geometrical thermodynamics properties.

## 1. Overview

One can use a descriptive nomenclature for things (“quantum wave equation”, for example) or an eponymous nomenclature (“Schrödinger equation”, for the same example). The latter fits a storytelling approach better, although one must always give the description somewhere! Here, for the convenience of readers of the “*Geometry in Thermodynamics III*” Special Issue, we briefly review the very large literature on the “Berry phase” analysis of a wide variety of complex systems. This is not an editorial summary of the Special Issue but rather an attempt to connect technical fields relevant to the Special Issue that appear almost entirely disconnected at present. In particular, one group of workers addresses “quantitative geometrical thermodynamics” as such [1], another addresses optical systems [2], and yet another addresses fast/slow dynamical systems [3]. Surprisingly, these are all formally related, and here we wish to give some sort of coherent overview of the field in general and these relations in particular. It is extraordinary how much work has been carried out in this general field, so this “review” is merely indicative; it is emphatically *not* exhaustive.

As Gu et al. [4] point out, “*when a classical or quantum system undergoes a cyclic evolution governed by slow change in its parameter space, it acquires a topological phase factor known as the geometric or Berry phase, which reveals the gauge structure in quantum mechanics*”. “Hannay’s angle” is the classical counterpart of this additional quantum phase [5] as is clear from the elegant treatment of a spinning top [6]. The quantum geometric phase and the classical Hannay’s angle really are closely related, an assertion confirmed by more recent work [7].

The Aharonov–Bohm effect (the strange phenomenon of a wave function phase shift caused by a field of zero magnitude) is by now well studied. It has even been observed as a phase shift in the proper timing of matter waves in response to a gravitational field (see Overstreet et al. [8], who have also helpfully summarised the field). Such phase shifts are known as “Berry phases” after Berry, 1984 [2] or “geometrical phases” (using Berry’s preferred descriptive nomenclature as he points to many illustrious contributors to the idea, including Pancharatnam [9]). Berry originally treated adiabatic systems but realised later that generalisation to the nonadiabatic case was “straightforward” [10]. This was also explained elegantly by Moore [11] in terms of the Floquet theorem (which solid-state physicists know as the Bloch theorem). Moore points out that the “Berry phase” has also been known as the “Aharonov–Anandan phase” as their treatment was actually the first to remove the adiabatic restriction [12], although it seems that the (nonadiabatic) Aharonov–Anandan phase may not be the same in the adiabatic limit as the (adiabatic) Berry phase [13]. The Pancharatnam phase (a special case of the Berry phase) is used in atom interferometry [14], a rapidly growing field. Ben-Aryeh reviewed both the mathematical formalism and applications to optical, atomic, neutron and molecular systems [15].

The canonical representation of the energy of a system classically uses the Hamiltonian formalism (where the energy balance is classically given by the Lagrangian). Roughly speaking, the Hamiltonian specifies total energy and the Lagrangian specifies the changes between potential and kinetic energy as the system evolves. The Schrödinger equation is most simply given as ***H***Ψ = *E*Ψ, where ***H*** is the Hamiltonian operator, Ψ is the “wavefunction” and *E* represents the eigenvalues of the operator, the point being that *real* eigenvalues are only guaranteed for an Hermitian operator. The topical review of Ghatak and Das [16] concentrates on non-Hermitian systems but underlines the general applicability of the ideas.

Again, Gu et al. [4] point out that “*in standard quantum theory, observable quantities are associated with Hermitian operators, and time evolution is generated by a Hermitian Hamiltonian, where the Hermiticity (or more precisely self-adjointness) of the Hamiltonian ensures both the real-valuedness of the energy spectrum and more importantly the unitarity*” (that is, losslessness or lack of dissipation) of time evolution. Therefore, it came as a surprise when Bender and Boettcher showed in 1998 [17] that non-Hermitian Hamiltonians can still possess real and positive eigenvalues. They claimed that the real-valuedness of the spectrum is due to parity time (PT) symmetry of the Hamiltonian. Gu et al. [4] also studied PT symmetric (or near-PT symmetric) systems, where the Hamiltonian is not Hermitian and where the Berry phases are nonadiabatic. This abstract approach to system classifications has been very fruitful, also resulting in an enormous literature.

The appearance of the PT symmetry was initially considered as a mathematically interesting finding with which many new Hamiltonians can be constructed (instead of just relying on Hermiticity). However, it turns out that non-Hermitian Hamiltonians are required for many cases, a certain class of which (so-called “pseudo-Hermitian”) continue to yield real eigenvalues. However, in the real world (the “general case”), the representation usually becomes complex.

Immediately, we see a prejudice against energy having an “imaginary” component. However, just because Descartes could not imagine that complex numbers could treat real things does not mean that a good representation of reality cannot rely on complex analysis. Indeed, complex analysis is now essential throughout the quantitative sciences.

**Figure 1:**
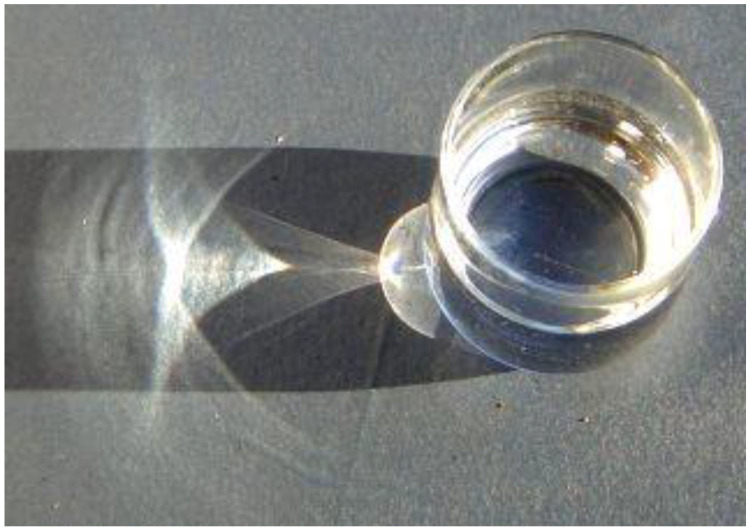
Caustics produced by a glass of water (wikimedia commons; © Heiner Otterstedt, Jan.2006; CCBY-SA3.0).

“Caustics” are common in simple optical systems (see Figure 1 and Berry and Upstill’s review [18]), and the engineering of high-quality optics goes to much trouble to eliminate them. It turns out that although such effects are ubiquitous, their physics is rather intricate, often turning on the geometry (or topology) of how the eigenvalues (and eigenfunctions) can coalesce. Such points of coalescence yield certain sorts of singularities in the formalism. These are usually called “exceptional points”, although Berry prefers “*nonhermitian degeneracies*” [19] saying, “*Nonhermitian models arise in physics as approximate descriptions of systems that are regarded as isolated, even though they exist in a larger environment*” [20] (strictly speaking, in reality, all systems are open; only idealised systems can be considered “closed”). It may be more correct to say that “*nonhermitian models have arisen in physics as approximations* …” but we must approximate to make any progress in physics at all; it is the *Hermitian* models that are an *idealisation* of reality! Berry points out that “caustics” and “nonhermitian degeneracies” are not directly related, for example, for diffraction in volume gratings with imaginary potentials “*diffraction is strongly affected by degeneracies of the non-Hermitian matrix generating the ‘Bloch waves’ in the grating … the asymptotic distribution of intensities among the diffracted beams … is … dominated by a single set of complex rays; this is very different from the semiclassical limit for transparent gratings, where the rays form families of caustics …*” [21].

However, the very general treatment of de Almeida and others [22,23] shows that phenomena that look like caustics from one point of view can be treated as non-Hermitian from another. In general, as Fan et al. comment, [24] “*the non-Hermiticity of a system implies that the energy bands are complex*” pointing out that the “*band gaps exhibit exceptional degeneracy that can induce rich new phenomena*”.

The quantum Hall effect (in which the Hall resistance is quantized, allowing redefinition of both the Planck constant and the elementary charge) and the quantum spin Hall effect (first observed in 2007 in HgTe quantum wells in CdTe) can both be understood in terms of the topological Chern classes [24]. These are topological invariants associated with vector bundles on a smooth manifold and can feasibly be expressed as polynomials in the coefficients of the curvature form. In particular, non-Hermiticity appears in nonequilibrium open systems with dissipative phenomena (or perhaps even the local nonconservation of probability associated with electron currents; see Chernyak et al. [25] and Modanese [26]). Recently, Fan et al. [24] proposed a complete topological classification of non-Hermitian systems, which generalises the 10-fold topological classification of Hermitian systems to a 38-fold classification scheme characterised by the winding number, vorticity, Berry curvature (the gauge field associated with the Berry phase), and Chern number of their quantum states. We should note that there is intense interest today in spintronic applications of the “topological Hall effect”. One useful example might be the very general discussion by Ishizuka and Nagaosa [27] of “*chiral spin-cluster scattering*”.

Fan et al. [24] note that “*the complex energy band gap for non-Hermitian systems*” can form a point or line gap that preserves its topological invariants under unitary transformations, but the energy band gaps close to form “exceptional points” (or lines; that is, non-Hermitian degeneracies) which act as reference points (or lines) associated with topological phases. They determine the Berry phase and the complex Berry curvature for the two-dimensional non-Hermitian Dirac model.

In Hermitian quantum systems, the real-valued Berry phase is known to be quantized in the presence of certain symmetries, and this quantized Berry phase can be regarded as a topological order parameter for gapped quantum systems. Tsubota et al. [28] establish that the complex Berry phase is also quantized in the systems described by a family of non-Hermitian Hamiltonians. It seems that the quantized complex Berry phase is capable of classifying non-Hermitian topological phases.

Gu et al. [4] propose a gauge transformation method to solve the time-dependent (periodically driven) Schrödinger equation whose Hamiltonian is a non-Hermitian (nonunitary) but PT symmetric operator. Quantum wave functions are obtained analytically along with the nonadiabatic Berry phase for the system. Then, the classical counterpart of the non-Hermitian Hamiltonian is solved in terms of the gauge transformation in classical mechanics. The explicit relation of Hannay’s angle and Berry phase is presented for the PT symmetric non-Hermitian Hamiltonian.

## 2. The Geometry of Irreversibility

The first obvious thing to say is that even in the adiabatic case, Hermitian and non-Hermitian Hamiltonian operators behave *qualitatively* differently, as explained in detail by Berry and Uzdin [29]. For an Hermitian operator, a degeneracy is a “diabolical” point (so-called after the “diabolo”, a juggling prop; see the helpful review by Yarkony [30]) at which the two eigenvalues are connected at the surface intersection *point*, which has the form of a double cone, and the two eigenstates are always orthogonal. By contrast, a degeneracy of a non-Hermitian operator is a branch point (commonly called an “exceptional point”), around which each eigenstate can “flip” into the other. Berry and Uzdin point out further subtle differences between the two cases: “*the eigenstate … occupation amplitudes [may] change drastically*” (and differently for the Hermitian and non-Hermitian cases) “*around the cycle*”. Such a behaviour discontinuity between idealised and real systems is widely observed: as a random example of this, in 1967 Charles Frank explained Hertzian fracture in an ideal case [31], but the formal description of the general case looks quite different [32].

It is important to recognise that the “*system records its history in a deeply geometrical way [involving] phase functions hidden in… regions which the system has not visited*”, as Berry points out [2] (recalling the nonlocality of the Aharonov–Bohm effect). This is intrinsic to the holomorphism and meromorphism (that is, piecewise holomorphic) central in the “quantitative geometrical thermodynamics” (QGT) of Parker and Jeynes [1]. This geometrical thermodynamics uses all the properties deriving from analytic functions, but Parker and Walker [33] explicitly demonstrate that “*Points of non-analyticity are inimical to any assumptions of ‘smoothness’, such that in general, when the system is allowed to dynamically evolve in time, assumptions of adiabaticity are not tenable, and one would expect the entropy to increase*.”

However, even though some systems are stable (that is, trivially reversible in time), their geometry can still encode the second law of thermodynamics. This is observed with Buckminsterfullerene [34], the alpha particle [35], and also the DNA double-helix backbone, whose chirality is a consequence of the second law [36]. That this encoding is not trivial is shown by the correct calculation not only of the matter radius of the alpha particle [35] but also of the Gibbs free energy difference between two forms of DNA [1]. This underlines the subtlety of the influence of irreversibility; apparently, the arrow of time may even be encoded into reversible systems.

As an example of an irreversible geometrical system, we consider Berry and Dennis’ detailed analysis of optical singularities in birefringent dichroic chiral crystals [37]. Optical systems are “simpler” than mechanical ones as there are fewer “practical details”; the proper treatment of friction and similar dissipative effects is notoriously complicated in mechanical systems. However, photons obey Maxwell’s equations, which are nice and “simple”, enabling the consideration of optical examples of irreversibility to be particularly informative. As with the case of caustic curves (see Figure 1), such complex examples are systematically avoided by the optics industry, which uses a variety of sophisticated methods to “keep things simple”. However, the effort of understanding the “ordinary” reveals the underlying physical complexities that may be grasped only by using sophisticated geometric representations.

Dichroic materials exhibit irreversibility as they absorb light. Formally, the reciprocal dielectric tensor ***η*** is defined by the relation between the electric field (***E***) and the displacement (***D***) vectors: ***E*** = ***η ∙ D***. Then, the symmetric part of ***η*** is the *anisotropy tensor* and the antisymmetric part is determined by the *optical activity vector*. For a dichroic crystal, the anisotropy tensor is complex (for a transparent crystal it is real). By formally considering all the possibilities (only touched on here), Berry and Dennis [37] show how the strange behaviour of such “simple” systems can be elegantly modelled in the general case.

**Figure 2:**
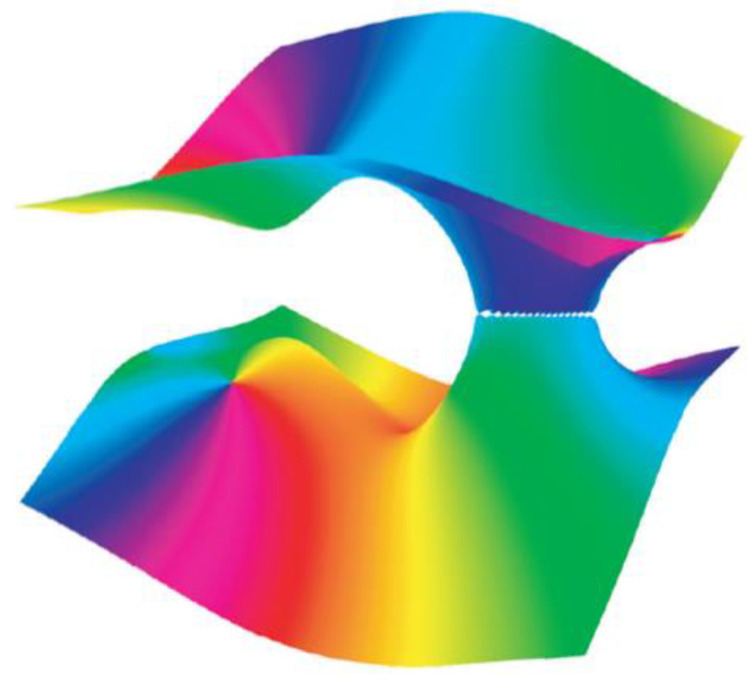
A representation of the (colour-coded) phase of the real part of the eigenvalues of *M* (Equation (1)) calculated in a certain simplification for chiral dichroic (absorbing) crystals (Figure 2c of Berry and Dennis [37], reproduced by permission).

In particular, the electric displacement vector ***D*** can be represented in a type of “Schrödinger equation”:

***M*** ∙ ***D*** = λ ***D*** (1)

where λ represents the eigenvalues of the matrix ***M***. This matrix is real symmetric for a transparent nonchiral crystal but complex non-Hermitian for a dichroic chiral crystal. Examination of Figure 2 illustrates discontinuities of various sorts in the phase solutions of this equation in a general case. It is the behaviour of these discontinuities that characterises the system behaviour.

More generally, the regular treatment for irreversible systems is to consider them as *perturbations* of reversible systems. The reason for this is that reversible systems are analytically more tractable than irreversible ones, and perturbation methods are well established. However, Roberts [38] quotes Lamarque et al. [39] saying, “*The main recognised drawback of perturbation methods is the absence of a criterion establishing their range of validity*.” Lamarque et al. demonstrate the existence of upper bounds on the range of validity, but Roberts also demonstrates the existence of lower bounds. Moreover, Roberts’ treatment is much more ambitious as he demonstrates (using a “backward modelling” method) the existence and construction of “centre manifolds” for a certain (rather wide) class of systems.

Centre manifolds are a systematic mathematical way to handle the properties of equilibrium points of dynamical systems that may be very complex, such as the tidal forces acting on the rings of Jupiter (see Zhang et al. [40] for an example of a Berry phase analysis of “tidal forces” in skin effects for certain metals). All fast/slow dynamical systems (such as the precession of a spinning top [41]; see also [6]) have stable and unstable “manifolds”, together with the “centre manifold”, which has important mathematical properties that govern the treatment of the whole system. Roberts [3] shows systematic ways of “untangling” such “fast/slow” systems. The point here is that “Hopf bifurcations” are ubiquitous features of “centre manifold” treatments, and these are closely related to “Berry phases”, as (for example) Ning and Haken [42] have shown in the context of dissipative optical systems.

Mecholsky [41] points out that, “As one of the simplest dynamical systems, the asymmetric top should be a canonical example to explore the classical analog of the Berry phase”. This comment is illustrated by a number of recent examples, such as Khatua and Ganesh’s [43] treatment of certain antiferromagnets, the development of the “kicked spinning top” by Mondal et al. [44] in the context of quantum spin models, and magneto-transport in spin-orbit coupled systems considered by Culcer et al. [45].

Roberts [38] treats the very general class of “nonautonomous dynamical systems”, whose dynamics is shown by Shao [46] to be deeply geometrical (in this context normally called “topological”). Such systems depend explicitly on time, including Brownian motion and other stochastic systems, and generalisations of them (see [3]).

“Topological entropy” is central to this description [46]. This idea was introduced by Adler et al. in 1965 [47] specifically to “*conform to previous work in ergodic theory*”. It turns out that an important issue is epistemological, that is, how much do we (and can we) know? The philosophical matters are treated rather generally by Jeynes et al. [48], but Roberts [38] points out that “*everything we know is only some kind of approximation, because we know that we do not know all the laws as yet*” (emphasis original, quoting Feynman). Roberts explains the “*classic distinction between backwards and forwards theory*”. The forwards theory “*asserts a centre manifold [that] exists but [which] often can only be known approximately … whereas the backwards approach asserts that there exists an exactly known centre manifold of a known system close to that specified. Forwards and backwards are complementary views*”. As we must approximate in any case, we can choose whether to approximate the centre manifold of the modelled system or to obtain the centre manifold of the approximated modelled system. It turns out that in the latter case, one can make full and fruitful use of the topology of the system.

## 3. Other examples of Berry phase treatments of Irreversibility

Grover et al. [49] observe an irreversible hysteretic anomalous Hall resistance in graphene (interpreted in terms of Chern number and Berry curvature), which they resolve at the nanoscale as domains of magnetisation. This is an interesting experimental example of an irreversible system interpreted in terms of Berry phases. Similarly, Jiang et al. [50] study an unusual chirality-dependent Hall effect in a tilted Weyl semimetal in the context of a treatment in terms of the Onsager–Casimir reciprocal relations, pointing out that these reciprocal relations imply the involvement of the Berry curvature.

The Berry curvature is also deeply implicated in a systematic treatment by Bian et al. [51] aimed at “*simulating photochemical and spin processes concomitantly*” relevant to “*spin−lattice relaxation dynamics*”. Culpitt et al. [52] point out that the Berry curvature is “*crucial*” in calculating the “*molecular dynamics in the presence of a magnetic field*”; it is “*used to calculate a screening force due to the electrons in the molecular system*”. The calculation of (nonadiabatic) Berry phases has been reviewed by Moore [53] and specifically for molecular systems by Mead [54].

Mesaros et al. [55] also consider the implications of the Onsager–Casimir reciprocal relations as applied in the case of dislocated graphene. They say that “*we stumbled on this story in trying to find out how to turn a topological phase that is most natural to graphene Dirac electrons into an observable quantity*”. In a very general treatment, they show that the effective conservation of some quantum numbers in certain sorts of quantum transport has “*the net effect that these coherent currents feel an “arrow of time” negating the Onsager relations associated with true equilibrium*”. The point is that dislocations are a topological (and nonlocal) feature of all atomic lattices, and they are also expected to be important in graphene. Saslow [56] also investigates “spin-pumping” using irreversible thermodynamics. He says, “*The irreversible nature of these quantities is not clear from Berry-phase approaches, where one employs current fluxes as primary variables, as if they were driven by a vector potential. However, in considering experimental quantities, which correspond to thermal averaging, these terms must be considered to be driven by non-uniform chemical potentials*”. This means that even “adiabatic spin-pumping” is irreversible.

Farag et al. [57] have investigated light–matter interactions in a “diabatic” representation, which is used in physical chemistry when the assumption that nuclear motion is negligible compared to that of electrons (known as the Born–Oppenheimer approximation) breaks down. They considered the coupling of a LiF molecule in an optical cavity “*to investigate the polariton quantum dynamics of the hybrid system*” with the general aim of finding new ways “*to manipulate photochemical reactivities of molecules with optical cavities*”.

Li [58] proposes “*geometric quantum computation” utilizing the “geometric phase depending only on the global geometrical feature of a cyclic evolution … for quantum control … maintaining the robustness against local disturbances during the evolution*” and demonstrates that robust operation is feasible for nonadiabatic operation. The point here, of course, is that adiabatic evolution is necessarily slow; a useful gate cannot be adiabatic.

Some extraordinary developments are taking place. A qubit can be constructed using the electronic levels of a nitrogen/vacancy (“NV”) centre in diamond (where the nitrogen is substitutional and associated with a vacancy). Using a static magnetic field aligned with the symmetry axis of the NV centre, both the NV electron spin and the ^14^N nuclear spin may be “*polarised by optical excitation*”. The qubit readout is from the fluorescence generated by the excitation. Ma et al. [59] demonstrate that this device is capable of pumping charge, and they interpret it as a “*generalised Thouless pump*” after Thouless [60], who first showed how such a charge pump would work. Takahashi et al. [61] explains that such “*geometrical pumping*” is a topological phenomenon to be interpreted in terms of the Berry phases of the system and that it is *not* restricted to adiabatic processes but is quite general.

Tokura and Nagaosa [62] helpfully review “*functional topological materials*” in terms of the Berry phase and the ubiquitous chirality of these materials. Parker and coworkers have treated chirality explicitly in the context of their geometrical thermodynamic formalism [1,34,36].

## 4. Conclusions

Physics has traditionally treated simplified systems, mainly because the simplified cases are the tractable ones. There has consequently been a bias in favour of either “engineering” the governing equations to only yield real solutions, which therefore have an “unambiguous” physical interpretation, or only considering the *real* part of complex solutions as the “real” solution desired. In particular, the Schrödinger equation has been thought to require an Hermitian Hamiltonian operator to guarantee real solutions, a move that essentially builds *reversibility* (and hence implies adiabaticity) into the representation. However, the intense recent interest in “Berry phase” analyses for a variety of systems has highlighted the complexity that obtrudes even in “simple” cases. It also highlights that the “Berry phase” analysis is applicable to “realistic” (essentially dissipative) systems and yields new insights into the underlying symmetries governing the physical behaviour of phenomena featuring a non-Hermitian Hamiltonian.

Reality is elusive, and the second law of thermodynamics guarantees that it is usually irreversible! The irreversible cases have usually been treated by perturbation methods as an “exception” of reversibility. Systematic approaches to irreversibility championed by Onsager and Prigogine and their schools have only slowly seeped into physics practice, and irreversibility is still seen as awkward to handle (and, in any case, less “fundamental” than reversibility). However, it is becoming clear that such an approach is wrong-headed. It is the *irreversible* case that is fundamental (just as it is the second law that is fundamental), and the Berry phase analysis makes an analytical treatment available that handles both reversible and irreversible cases on the same basis.

In this review, we have tried to explain how such an approach (which is deeply geometrical) is connected to other more explicitly thermodynamic approaches (also deeply geometrical). This is of great importance as the geometry of a system embodies its nonlocal properties, which are usually expressed in terms of the variational principles (least action, maximum entropy, etc.). Reality is elusive precisely because it can be handled correctly only in a fully complexified theoretical framework in which eigenvalues are not real in the general case. Recent work [63] has shown that for a more satisfactory and unified explanation of many physical phenomena, even the description of time should be complexified.

We need to become more aware of the variety of sophisticated analytical tools that are becoming available to consistently treat an ever wider and more thermodynamically diverse set of (mostly irreversible) physical phenomena and of the connections between them.

Robert Zwanzig introduced the “projection operator” method in 1960 [64] when he “*presented, for the first time, a formalism to calculate dynamic physical quantities without resort to perturbation theory … this trailblazing work is truly a landmark in the history of irreversible statistical mechanics*” [65]. The eponymous “Mori–Zwanzig” transformations have a close formal (and also, one suspects, physical) relation to Roberts’ formalism [38]. Here, we have sketched various apparently disparate analytical approaches to irreversible phenomena for which one can demonstrate a surprisingly close relation. The difficulty is that the relations are most clearly expressed in terms of the “Berry phase”, which is not a simple idea, being an abstraction of an abstraction. Here, we have described this variety in what may appear to be a “superficial” way, that is, involving almost no explicit formalism. However, we hope that workers may be alerted to cognate parallel approaches and that this new awareness will be fruitful.

## Data Availability

No new data were created or analyzed in this study. Data sharing is not applicable to this article.
